# *Duguetia furfuracea* (A.ST. Hil.) Saff.: Neuroprotective Effect on Chemically Induced Amnesia, Anxiolytic Effects and Preclinical Safety Evaluation in Mice

**DOI:** 10.3390/biology13120981

**Published:** 2024-11-27

**Authors:** Maiara Fava de Souza, Jéssica Maurino dos Santos, Sidney Mariano dos Santos, Pedro Cruz de Oliveira Junior, Janaine Alberto Marangoni Faoro, Arielle Cristina Arena, Lívia Trippe Nagaoka, Gisele de Freitas Gauze, Rodrigo Juliano Oliveira, Matheus Henrique Barbim Rech, Rosilda Mara Mussury Franco Silva, Anelise Samara Nazari Formagio

**Affiliations:** 1 College of Health Science, Federal University of Grande Dourados, Dourados 79804-970, MS, Brazil; maiarafava_777@hotmail.com (M.F.d.S.); jessicamaurinodossantos@gmail.com (J.M.d.S.); sidneysmariano@gmail.com (S.M.d.S.); janaine_dec4@hotmail.com (J.A.M.F.); 2 College of Biological and Environmental Sciences, Federal University of Grande Dourados, Dourados 79804-970, MS, Brazil; pedrojuniorbiologo@gmail.com (P.C.d.O.J.); mussuryufgd@gmail.com (R.M.M.F.S.); 3 Institute of Biosciences of Botucatu, Department of Structural and Functional Biology, University Estadual Paulista—Botucatu (UNESP), São Paulo 18618-689, SP, Brazil; arielle.arena@unesp.br (A.C.A.); livia.t.nagaoka@unesp.br (L.T.N.); 4 Department of Chemistry, State University of Maringá UEM, Maringá 87020-900, PR, Brazil; giselegauze@yahoo.com.br; 5 Centro de Estudos em Células-Tronco, Terapia Celular e Genética Toxicológica (CeTroGen), Faculdade de Medicina (FAMED), Universidade Federal de Mato Grosso do Sul (UFMS), Campo Grande 79070-900, MS, Brazil; rjo.rodrigojulianooliveira@gmail.com (R.J.O.); matheus_rech@ufms.br (M.H.B.R.)

**Keywords:** “araticum seco”, AChE, anxiety, dementia, scopolamine

## Abstract

*Duguetia furfuracea*, “araticum-seco”, is known to contain several bioactive compounds that can mitigate oxidative stress and act on the central nervous system (CNS). This effect is partly attributed to its potent antioxidant and acetylcholinesterase (AChE) inhibitors. In this study, the effects were explored of the methanolic extract (MEDF) and alkaloid fraction (AFDF) of *D. furfuracea* (leaves) on cognitive behaviors in male mice with scopolamine (Scop)-induced cognitive impairment and biochemical parameters. Additionally, anxiolytic behavior, subacute toxicity, molecular docking and antioxidant activity were reported. MEDF (30, 100 or 300 mg/kg) or AFDF (30 mg/kg) were orally administered for 16 days and Scop (intraperitoneally, i.p.) between days 11 and 16. The anxiolytic behavior (open field test and marble burying) in healthy mice, and the Scop-induced memory impairment (object recognition test and Morris water maze (MWM)) were assessed, and the biochemical parameters (malondialdehyde (MDA) and AChE levels) were measured after euthanasia. The subacute toxicological impact of MEDF was assessed in female Swiss mice for 28 days. MEDF and AFDF were available for the DPPH, ABTS and β-carotene/linoleic acid models. The results revealed that MEDF and AFDF exhibit anxiolytic effects and significantly alleviated Sco-induced memory impairment, inhibited AChE in the cortex (40%) and MDA (51.51%) levels. Reticuline was reported in AFDF and molecular coupling with AChE involves link-type hydrogen bonds and van der Waals interactions. MEDF exhibited antioxidant capacity (DPPH, IC_50_ = 18.10 ± 1.70 µg/mL; ABTS, IC_50_ = 10.41 ± 1.69 µg/mL). MEDF did not reveal signs of toxicity. In conclusion, *D. furfuracea* shows promise in mitigating scopolamine-induced memory deficits, potentially because it inhibits AChE activity, reduces MDA levels, and enhances antioxidant activities.

## Simple Summary

*Duguetia furfuracea* is distributed in the Brazilian Cerrado and is used in popular medicine to manage various diseases. Although it has demonstrated anti-inflammatory effects, there is currently no scientific evidence demonstrating its neurobehavioral actions, given that this species contains alkaloids as its chemical constituents. In this research, the potential neurobehavioral effects of methanolic extract and an alkaloid-rich fraction of *D. furfuracea* in mice were explored. Molecular coupling analysis revealed that reticuline (alkaloid) can substantiate its biological activities.

## 1. Introduction

Neurodegenerative disorders, such as Alzheimer’s disease (AD), one of the most common forms of dementia in the elderly population, are characterized by cognitive function loss and progressive memory impairment (related to reasoning and learning capacity), which result in a detrimental impact on daily activities. The treatment for this disease primarily involves mitigating its symptoms, and the approach in therapy involves enhancing cholinergic transmission, which serves as the primary strategy. This cognitive impairment is predominantly linked to reductions in cholinergic neurotransmission systems and neuronal dysfunction, with decreased levels of brain acetylcholine (ACh), the overexpression of acetylcholinesterase (AChE) and increased oxidative stress constituting some of the main features of the disease [1,2,3]. Therefore, medicinal plants are promising sources of future therapeutics for amnesia and other oxidative stress-related conditions.

*Duguetia furfuracea* (A. St.-Hil.) Saff., Annonaceae, popularly known as “araticum-seco”, “ata brava” or “marolo”, is a native shrub of the Cerrado (3 m) and is found in the Brazilian state of Mato Grosso do Sul [4,5]. Leaves (infusion) are used in popular medicine to treat pain and rheumatism [6,7]. The biological activities of *D. furfuracea* (leaves) include antifungal and antioxidant effects [8]. Studies carried out by our research group demonstrated in vitro anticholinesterase and antiproliferative activity [9] and antirheumatic and anti-inflammatory potential in mice, and oxaporphine alkaloids could be responsible, at least in part, for the observed effects [10]. However, the toxicity of *D. furfuracea* has been further investigated. Extracts derived from the leaves of this species have been reported to have cytotoxic effects on pregnant rats [11,12], as well as antigenotoxic and anticytotoxic activities in bacterial and murine assays [13]. Additionally, impairments in negative geotaxis behavior, indicative of intermediate toxicity, were observed in *Drosophila melanogaster* [14].

Polyphenolic compounds (catechin, gallic acid, quercitrin rutinellagic acid, caffeic acid and isoquercitrin) and alkaloids, mainly aporphine alkaloids, a subclass of isoquinoline (dicentrinone, liriodenine, lanuginosine, atherospermidine, and reticuline), are present in *D. furfuracea* [15,16,17,18,19]. Alkaloids, such as reticuline, have been scientifically studied for their ability to stimulate the central nervous system (CNS) effects and block dopaminergic receptors [20]. Our previous research with *Unonopsis stipitate* revealed that the leaves of members of the Annonaceae family in the Amazon region are used for the treatment of cognitive disorders, and reticuline was shown to be alkaloid active [20]. These alkaloids (aporphine and isoquinoline) showed significant activity related to AD, and most of them moderately inhibited cholinesterases.

Scopolamine (Scop)-induced cognitive impairment in rodents is a common model used in neuroscience and pharmacology to study cognitive decline and explore potential treatments for memory disorders such as AD. Scop is a nonselective, competitive, postsynaptic muscarinic receptor inhibitor that can lead to cognitive impairment in both rodents and humans. The mechanism of action involves reducing the impact of acetylcholine in the CNS. Acetylcholine is crucial for learning and memory, so its inhibition can lead to cognitive deficits [21,22]. In this context, alkaloids, such as galantamine extracted from *Galanthus nivalis*, can interact with AChE. Like physostigmine, galantamine is a reversible inhibitor of AChE. It enhances cholinergic neurotransmission and is used to treat mild to moderate AD.

Owing to the rich phytochemical composition of *D. furfuracea*, particularly alkaloids, and the presence of reticulin, which affects the CNS, we hypothesize that the extract of *D. furfuracea* and the alkaloid-fraction improve impairments in learning, memory and object recognition in this rodent model. Thus, in this study, the aim was to investigate the effects of the methanolic extract (MEDF) and alkaloid fraction (AFDF) from *D. furfuracea* leaves on scopolamine-induced amnesia in mice, with a focus on the neuroprotective effects and the ability to prevent emotional and spatial memory (object recognition and Morris water maze (MWM)) and biochemical parameters (AChE and malondialdehyde (MDA)). In addition, AFDF was analyzed via ultra-high-pressure liquid chromatography–high resolution mass spectrometry (UHPLC–MS/MS) and the molecular coupling of reticuline with AChE. Additionally, anxiolytic-like behavior (open field and marble burying) in healthy male mice, subacute toxicity after daily oral treatment with MEDF for 28 days in female Swiss mice, phenolic and polyphenolic compounds and antioxidant activity were reported.

## 2. Materials and Methods

### 2.1. Material Vegetal

The aerial parts (leaves and thin branches) of *D. furfuracea* were collected in May 2022 in Dourados, Mato Grosso do Sul, Brazil (22°48′53″ S and 54°44′31″ W). Botanical identification was performed by Dr. Zefa Valdevina Pereira and a voucher specimen (8979) was deposited in the Herbarium of this University (UFGD, Brazil). Authorization for the access to and study of Brazilian genetic heritage samples was obtained from the National System for Genetic Heritage and Associated Traditional Knowledge Management (SisGen—A51F665).

### 2.2. Drugs and Chemicals

Scopolamine hydrobromide, acetylthiocholine iodide, 2,2-diphenyl-1-picrylhydrazyl (DPPH) and 5,5′-dithiobis [2-nitrobenzoic acid] (DTNB) were acquired from Sigma-Aldrich (St. Louis, MO, USA). Bovine serum albumin–lyophilized powder was also obtained from Sigma-Aldrich (St. Louis, MO, USA). All the other materials were of the highest available quality.

### 2.3. Extraction and Fractionation of the Methanolic Extract of D. furfuracea

Leaves (4.15 kg) of *D. furfuracea* were dried by circulation at 40 °C for 7 days and then powdered (1.10 kg). Some of the air-dried leaves (800 g) were extracted by macerating for 25 days with methanol (MeOH, 3 L) at room temperature (25–28 °C), followed by filtration with a solvent (MeOH) and renewal every 2 days. Evaporation of the MeOH mixture under reduced pressure at 65 °C and subsequent lyophilization with an Alpha 1–2 LD Plus system with Christ equipment (Lyophilizer Christ, Osterode am Harz, Germany) and vacuum parameters of 0.045 mbr and a temperature of −42 °C were used to produce the methanolic extract (MEDF) (87.70 g), which was stored under refrigeration at 4 °C for further testing. Part of this extract (40 g) was treated with aqueous HCl (1 N, 100 mL), and the acidic solution was extracted with dichloromethane (CH_2_Cl_2_, 300 mL). The aqueous phase was basified with NH_4_OH to a pH of 9–10 and extracted with CH_2_Cl_2_ (600 mL). The organic fractions were combined, and the solvent was evaporated to yield the alkaloid fraction (AFDF, 444.80 mg). Visualization of the compounds (preferably alkaloids) on thin-layer chromatography (TLC) eluents (CHCl_3_: MeOH at 70%) was accomplished by UV irradiation at 254 and 366 nm or by Dragendorff’s reagent to confirm that AFDF was positive for alkaloids.

### 2.4. Content Constituent’s

The content of phenolic and polyphenolic compounds was determined in MEDF. The content of phenolic compounds was determined using the Folin–Ciocalteu reagent, with a different concentration of MEDF (1 mg/mL) [23]. The absorbance was measured at 760 nm using a spectrophotometer (Model Bel UV-M51, Monza (Milano), Italy) and a standard curve of gallic acid (1.06 mg/mL) was used, y = 5.2407x + 0.27247, with a correlation coefficient of R^2^ = 0.985. The concentration was expressed in terms of gallic acid equivalents (GAE) in mg/g of extract. To determine the flavonoid and flavonol contents, solutions were used of the MEDF (2 mg/mL) and a standard curve of quercetin (1.02 mg/mL) (y = 10.221x + 0.082; R^2^ = 0.997), in absorbance 440 nm [23]. The results were expressed as quercetin equivalents (QE) in mg/g of the extract. Condensed tannins in the MEDF were mixed with 5 mL vanillin-HCl (8% conc. aq. HCl and 4% vanillin in methanol), absorbance was measured at 510 nm, and a standard curve of catechin (10.05 mg/mL) (y = 0.77087x + 0.0504; R^2^ = 0.997) was obtained [23]. The results were expressed in catechin equivalents (CE) in mg/g of extract. All assays were performed in triplicate.

### 2.5. Chemical Analysis of AFDF by UHPLC—MS/MS

The AFDF was solubilized in methanol-acetonitrile (1:1, v/v), due to solubility, mobile phase compatibility, reverse phase chromatography, viscosity and ionization MS, at a concentration of 0.5 mg/mL and centrifuged (1200× *g*, 5 min), and the supernatant was analyzed, with a UHPLC system (Shimadzu, Nexera X2, East Lyme, CT, USA) equipped with a CBM-20A system controller, two LC-30AD pumps, a CTO-30A column oven and a SIL-30AC autosampler coupled to an HRMS system (QTOF Impact II; Bruker Daltonics Corporation, Fremont, CA, USA) equipped with an electrospray ionization source. The capillary voltage was operated in positive ionization mode and was set to 4500 V with an endplate offset potential of −500 V. The dry gas parameters were set to 8 L/min at 200 °C with a nebulization gas pressure of 4 bar. Chromatographic separation was performed using a C18 column (75 × 2.0 mm i.d.; 1.6 μm Shim-pack XR-ODS III, Torrance, CA, USA), which offers high efficiency on particles, and analyses of polar and moderately polar compounds that differ in hydrophobicity. The gradient mixture of solvents A (H_2_O) and B (acetonitrile with 0.1% formic acid; v:v) was as follows: 5% B 0–1 min, 30% B 1–3 min, 95% B 3–12 min, maintained at 95% B 12–16 min, and 5% B 16–17 min. The flow rate was 0.2 mL/min, the column temperature was 40 °C and the injection volume was 3 µL. The data were processed with Bruker Compass Data Analysis 4.3^®^ software (Bruker Daltonics, Bremen, Germany). Data were collected from m/z 50–1300 with an acquisition rate of 5 spectra per second, and the ions of interest were selected by automatic MS/MS scan fragmentation and the identification was confirmed by comparing it to the published reports or databases such as ACD Lab, Inc. (Toronto, ON, Canada), and by analyzing the ion fragmentation patterns. The compounds were proposed based on a bibliographic review of the genus and species, as well as the error value of the mass.

### 2.6. Antioxidant Activity

#### 2.6.1. Free Radical-Scavenging Activities—DPPH and ABTS Reagents

The antioxidant activity was determined via the stable free radical 2,2-diphenyl-1-picrylhydrazyl (DPPH) scavenging method [23,24]. Different concentrations (0.01, 0.25, 0.5, 1, 5, 25, 75, 125 and 250 μg/mL) of MEDF or AFDF were added to 3 mL of DPPH (0.1 mM, methanol), and after 30 min (room temperature in the dark), the absorbance was measured at 517 nm.

The capture of the radical cation 2,2′-azinobis-3-ethylbenzothiazoline-6-sulfonic acid (ABTS+) by MEDF and AFDF was determined [23]. For the ABTS+ solution (7 mM ABTS and 140 mM) of potassium persulfate in ethanol, an absorbance of 0.7 ± 0.05 nm at 734 nm was obtained. The ABTS+ solution (1.980 µL) was carefully mixed with 30 µL of each concentration of the test sample and shaken to homogenize. The samples were allowed to react for 6 min and to rest in the dark, and the absorbance at 734 nm was measured with a spectrophotometer.

BHT samples with the same concentrations were used as standards, and the solutions without added extracts (in methanol) were used as controls (blanks). The test was carried out in triplicate. The percentage of inhibition was calculated according to the following equation: % I = (1 − sample absorbance/control absorbance) × 100, and the antioxidant agent’s capacity to scavenge 50% of the DPPH free radicals present in the solution (IC_50_) was determined.

#### 2.6.2. Linoleic Acid Peroxidation—β-Carotene/Linoleic Acid

A total of 20 mg of β-carotene solubilized in 10 mL of chloroform was used. For the emulsion, 20 μL of linoleic acid, 1 mL of β-carotene-chloroform solution (2 mg/mL) and 200 μL of Tween 40 were used [23]. After evaporation of the chloroform, 50 mL of distilled water was added under stirring (emulsion), and subsequently, 5 mL of this emulsion was added to different concentrations of the MEDF and AFDF (1, 25, 50, 100, 175 and 250 μg/mL). The mixture was placed in a water bath (~50 °C) (105 min), and oxidation was monitored by absorbance (measured at 470 nm for 105 min) at 15 min intervals. BHT was used as a standard control at the same concentrations as the samples. The solutions without added extracts were used as controls (blank). Antioxidant activity, measured as the percentage of bleaching inhibition, was determined via the formula %AA = 100 − [(Ai − At)/(A’i − A’t) × 100]. Ai = initial absorbance of the sample, At = absorbance after 100 min of incubation at 50 °C, A’i = initial absorbance of the control and A’t = control absorbance after 100 min of incubation at 50 °C. The results were reported as the IC50 values. The assay was performed in triplicate.

### 2.7. Experimental Animals

Male and female Swiss mice (25–32 g) were used, obtained from the UFGD Central Animal Facility, and all the doses were adjusted according to the weight of each animal. Experimental procedures were in accordance with the Ethical Committee in Animal Experimentation of UFGD (35/2022), were kept in plastic cages lined with pine shavings, provided with ad libitum food and water under a 12 h light/dark cycle at environmental conditions (25 ± 2 °C). The handling of the animals, before and during the experiments, followed the principles of animal experimentation ethics (CONCEA—National Council for Control of Animal Experimentation).

### 2.8. Subacute Oral Toxicity

The Organization for Economic Cooperation and Development (OECD) guideline 407 has established parameters for toxicity studies involving rodents. This guideline, which was referenced in a previous study [25], specifies the use of rats or mice for both acute and subacute protocols; in carrying out the project, we opted for the use of mice instead of rats because they are easier to manage. The female mice were divided into five groups (n = 6 per group), resulting in a total of 30 mice. The mice were treated for 28 consecutive days, as follows: control with 0.9% saline solution (10 mL/kg, p.o.), vehicle control with Tween 80 (5%) (dissolved in 0.9% saline solution, 10 mL/kg, p.o.) and MEDF (30, 100 or 300 mg/kg, p.o.).

Physiological data (weight, water intake and food consumption) were observed and recorded daily, along with any abnormal behavioral changes throughout this study. At the end of the test period (28 days), all the animals were fasted for 12 h and were intraperitoneally anesthetized with a combination of ketamine and xylazine (25 mg/kg and 10 mg/kg, respectively), and blood collection, including hematological (EDTA-coated tubes) and biochemical (dry tubes) parameters, was performed via cardiac puncture for laboratory evaluation. A macroscopic evaluation of the organs (brain, heart, lungs, liver, spleen and kidneys) was subsequently conducted, with each organ being removed and cleaned in saline solution to determine the relative weight [25]. Additionally, a sample from each organ was collected and preserved in Alfac solution (85% ethanol, 10% formaldehyde, 5% glacial acetic acid). The samples were embedded in paraffin, sectioned at 5 μm, stained with hematoxylin and eosin and examined via light microscopy [26].

### 2.9. Neurobehavioral Tests

**Figure 1:**
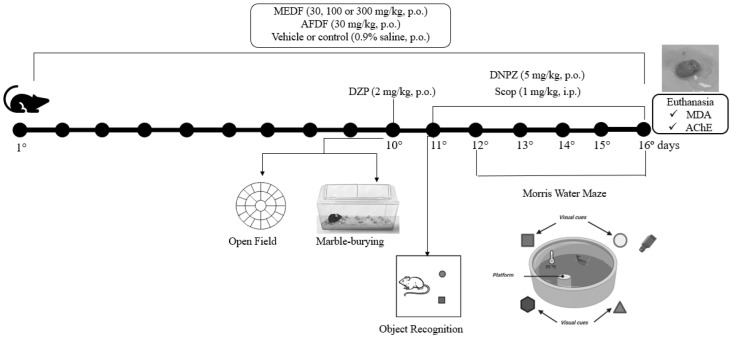
Experimental design of behavioral tests that assess anxiety, learning and memory in mice and biochemical parameters (MDA and AChE).

This experiment (Figure 1) was conducted using male mice (n = 8) randomly distributed into 8 groups, as follows: (I) -Vehicle (0.9% saline solution, p.o.), (II)—MEDF (30 mg/kg, p.o.), (III)—MEDF (100 mg/kg, p.o.), (IV)—MEDF (300 mg/kg, p.o.), and (V)—AFDF (30 mg/kg, p.o.) were dissolved in 0.9% saline solution and administered daily for 16 consecutive days; (VI)—diazepam (DZP) (2 mg/kg, p.o.) was administered only on the 10th day; and (VII)—donepezil (DNPZ) (5 mg/kg, p.o.) and (VIII)—scopolamine (Scop) (1 mg/kg, intraperitoneally, i.p.) were also dissolved in 0.9% saline solution and administered daily for 6 days (11th to 16th days). Only the vehicle group (I) did not receive Scop.

To evaluate the dose–response effects, three doses of MEDF (30, 100 and 300 mg/kg) were used, and selected on the basis of previous studies by our research group with *D. furfuracea* [10]. Considering that the lowest dose of MEDF (30 mg/kg) was effective for some anxiety, learning and memory parameters, and research ethics limits the use of animals, this dose was used for alkaloid fraction AFDF (30 mg/kg). The animals only received the treatment after adaptation (one week). At the end of the behavioral assessment, the mice were sacrificed for biochemical assays to assess MDA levels (brain homogenate) and AChE activity (cortex and hippocampus).

### 2.10. Anxiety-like Behavior

#### 2.10.1. Open Field Test

On the 10th day, after 1 h of treatment with MEDF (30, 100 or 300 mg/kg, p.o.), AFDF (30 mg/kg, p.o.), control positive DZP (2 mg/kg, p.o.) or vehicle (0.9% saline, p.o.), the mice were placed in the center of the open field apparatus and assessed individually for 5 min. Subsequent behavioral changes, including the time spent in the central zone, number of instances of self-grooming and number of rearing events [27], were recorded via software (ANY-Maze, 7.20). The base of the apparatus was cleaned with 70% alcohol to remove odors.

#### 2.10.2. Marble Burying

On the 10th day, 1 h after the administration of MEDF (30, 100 or 300 mg/kg, p.o.), AFDF (30 mg/kg, p.o.), DZP (2 mg/kg, p.o.) or vehicle (0.9% saline, p.o.), each mouse was placed in a polypropylene box (26.5 × 42 × 15 cm), with the bottom covered with a layer of sawdust (5 cm thick). Twenty glass marbles (1 cm in diameter) were placed in four rows, and the total number of marbles that were completely buried or two-thirds buried was recorded over a period of 5 min [28].

### 2.11. Memory and Learning—Scopolamine Induction

#### 2.11.1. Object Recognition

On the 11th day, after treatment with MEDF (30, 100 or 300 mg/kg, p.o.), AFDF (30 mg/kg, p.o.), control (0.9% saline, p.o.) or DNPZ (5 mg/kg, p.o.), the mice received Scop (1 mg/kg, i.p.) 30 min before the test. Posteriorly, the mice were placed in a box containing two identical objects and allowed to explore them for 10 min. After 1 h of training, the test was carried out, with the mice placed in a box that contained the familiar object and a new object, and exploration was recorded for 5 min. Object exploration was defined as active, direct interaction (e.g., touching and smelling) with the object or directing the nose toward the object from a distance of 2 cm. Exploration time was recorded via video tracking software (ANY-Maze). The performance was calculated on the basis of the time spent exploring the new object (TN) and the time spent exploring the old object (TO) [29].

#### 2.11.2. Morris Water Maze (MWM)

On the 11th to 16th days, after treatment with MEDF (30, 100 or 300 mg/kg, p.o.), AFDF (30 mg/kg, p.o.), control (0.9% saline, p.o.) or DNPZ (5 mg/kg, p.o.), the mice received Scop (1 mg/kg, i.p.) and were subjected to the Morris water maze (MWM) test. The 12th to 15th days were training sessions, and the 16th day was the test itself [30]. The labyrinth consisted of a circular pool (diameter: 180 cm, depth: 60 cm) filled with water (25 ± 1 °C) to a depth of 35 cm and was divided into four identical quadrants containing clues on the pool wall. In the central area of one of the quadrants, the circular platform (10 cm in diameter) was positioned and hidden 1 cm below the water surface. During training sessions (4 days), each mouse could swim for 60 s to reach the escape platform. There was a rest period of 30 s between each attempt (4 in total). If the mouse was unable to locate the platform within the stipulated time, it was guided to its location and remained there for 20 s. In the training session, the time spent detecting the escape platform was considered a learning process (memory acquisition) determined by software (ANY-Maze). On the 16th day, the platform was removed, and the animals were observed for 60 s. Memory consolidation and motor performance were assessed on the basis of the acquisition index, which was determined as the average escape latency time for the animal to find the hidden platform(s). In addition, parameters such as time in the platform zone (s), number of entries into the target quadrant, and distance traveled to the first entry into the target quadrant (m) were recorded.

### 2.12. Biochemical Parameters

#### 2.12.1. Malondialdehyde (MDA)

On the 16th day, the mice were euthanized to assess MDA activity via brain homogenates treated with MEDF (30, 100 or 300 mg/kg), AFDF (30 mg/kg) or Scop (1 mg/kg). The brain (maintained in phosphate buffer of a pH of 7.4 in a volume 4 times greater than the weight) was homogenized and centrifuged at 3000 rpm for 10 min, the supernatant was diluted (3 times its volume), trichloroacetic acid (10%) was added and the mixture was centrifuged at 3000 rpm for 20 min. Four milliliters of the supernatant was removed, the mixture was incubated in a water bath at 37 °C for 1 h, 0.67% (1 mL) thiobarbituric acid was added, and the mixture was incubated again in a water bath (100 °C) for 15 min [31]. The developed pink chromogen was read at 532 nm. The test was carried out in triplicate, and the results were calculated with the following equation: (I%) = [(control + Scop absorbance − sample absorbance)/absorbance of the control + Scop)] × 100.

#### 2.12.2. AChE Activity

After the brain structure was removed, the cerebral cortex and hippocampus were separated, homogenized in 0.01 M potassium phosphate buffer solution (pH 7.4) at a concentration of 5% w/v, cooled to 4 °C and centrifuged at 11,000 rpm for 10 min. Subsequently, the supernatant was collected for protein measurement. The protein concentration of the samples was determined via the Bradford reagent (Sigma-Aldrich), with a bovine serum albumin curve (BSA; Sigma-Aldrich) used as a standard. With 10 μL of each concentration and 200 μL of Bradford reagent added, the reaction was determined at 595 nm after 10 min at room temperature. Afterward, to determine AChE activity, 100 μL of buffered Ellman’s reagent (0.01 M DTNB), 150 μL of potassium phosphate buffer (0.1 M, pH 7.4) and 25 μL of the brain structure were added, and this mixture was allowed to react at room temperature for 5 min [32]. Then, 20 μL of acetylthiocholine iodide solution (0.075 M) was added, and the absorbance was measured at 415 nm for 7 min at intervals of 30 s. All the reactions were performed in triplicate, and the enzymatic activity was expressed as μmol ASCh/h/mg of protein.

### 2.13. Docking Simulations

The crystal structure of mAChE (acetylcholinesterase–*Mus musculus*) complexed with choline (code ID: 2HA3) was obtained from the Protein Data Bank, and molecular docking studies were performed via Molegro Virtual Docker v6.0 [33]. The structure of reticuline (PubChem CID: 439653) was obtained from PubChem (https://pubchem.ncbi.nlm.nih.gov/, accessed on 14 May 2023). All water molecules and ions were removed from the structures of mAChE. The docking protocol was carried out using the Moldock score as a scoring function and the Moldock optimizers a search algorithm, with the search sphere radius set to 11 Å around the catalytic site and all other options set to the default value [34]. The redocking simulations were repeated five times, and the results were reproducible. The results were ranked via Rerank scores, and the enzyme–ligand interactions were analyzed via CCP4mg molecular-graphics software, 2.10.11 [35].

### 2.14. Statistical Analysis

The data are presented as the mean ± standard error (SEM). Significant differences were determined via analysis of variance (ANOVA) followed by the Tukey test for neurobehavioral and Student–Newman–Keuls test for toxicity (GraphPad Prism Software 5.0). Differences were considered significant at *p* < 0.05.

## 3. Results

### 3.1. Chemical

The MEDF presented the highest values of condensed tannins (650.64 mg CE/g extract), followed by total phenols (590.27 mg GAE/g extract). Flavonoids (27.73 mg QE/g of extract) and flavonols (32.13 mg QE/g of extract) were present at low concentrations.

**Figure 2:**
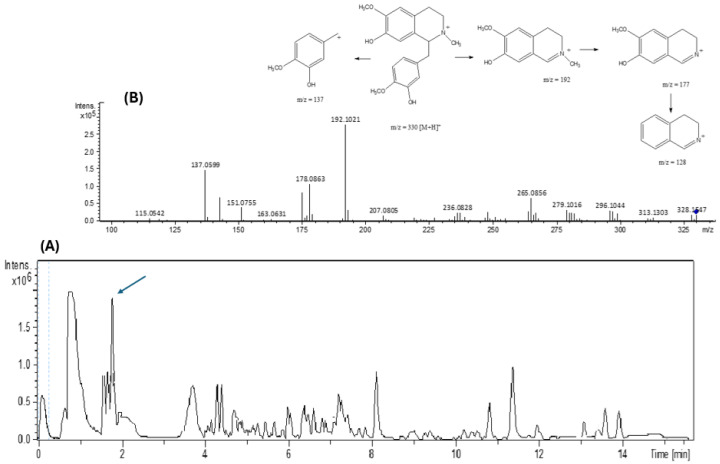
LC–MS (**A**) and (**B**) MS spectra/fragmentation at 1.84 min of AFDF.

Dragendorff’s reagent was used to confirm that AFDF was alkaloid-positive, as orange-colored halos, with a retention index R_f_ = 0.23 (Supplementary Materials). AFDF was carried out via LC–MS/MS (Figure 2A,B). The metabolite identified was reticuline (rt = 1.84 min), corresponding to m/z 330.1701 (Figure 2A).

### 3.2. Antioxidant Activity

The results showed that compared with BHT, MEDF has potent antioxidant activity by reducing the DPPH radical (IC_50_ = 18.10 ± 1.70 µg/mL) and ABTS (IC_50_ = 10.41 ± 1.69 µg/mL) concentrations (Table 1).

### 3.3. Toxicity Studies

#### Subacute Oral Toxicity

**Figure 3:**
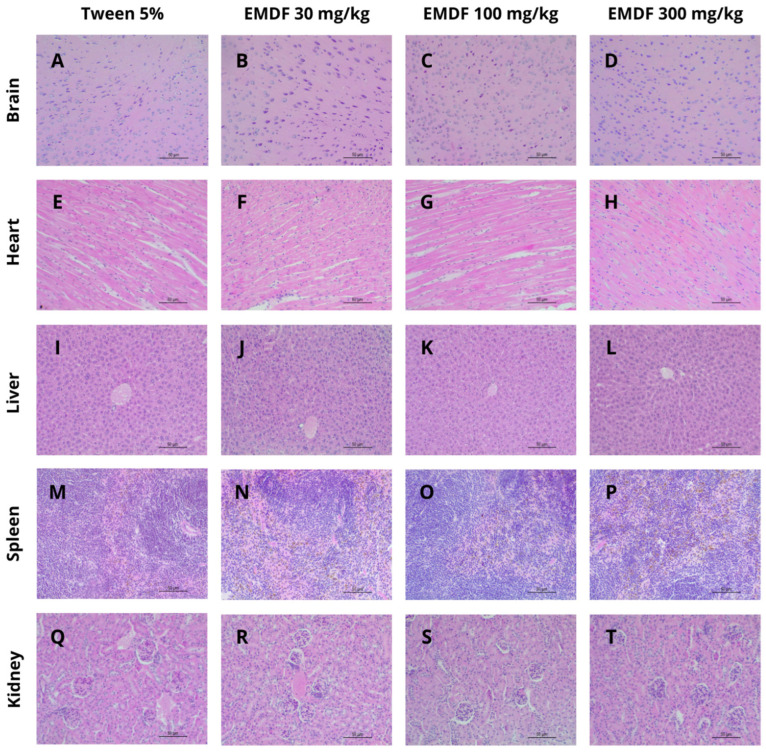
Photomicrography of organs stained with H&E in 40× magnification. (**A**–**D**): brain sections from cerebral cortex; (**E**–**H**): muscle fibers in the cardiac muscle; (**I**–**L**): normal liver sections evidencing the central vein; (**M**–**P**): sections of spleen samples; (**Q**–**T**): kidney sections with normal architecture of glomeruli.

No clinical signs of toxicity were observed in the mice after treatment with MEDF (30, 100 or 300 mg/kg) in the subacute toxicity test. MEDF did not promote any change in the weight, hematological or biochemical values or morphology of the analyzed organs, as indicated in Table 2 and Table 3. Analysis of the microscope slides prepared from the collected organs revealed that the treated groups did not differ from the control groups, even at the cellular level (Figure 3).

### 3.4. Anxiolytic Activity—Open Field Test and Marble Burying Test

Compared with the vehicle, the oral administration of MEDF (300 mg/kg) and AFDF (30 mg/kg) caused an increase in time in the central zone of 76% (*p* < 0.05) and 80% (*p* < 0.01), respectively, and a decrease in “rearing” lifts of 67% and 69% (*p* < 0.01), respectively, in the open field test (Table 4). No significant differences were detected between any of the treatments (MEDF and AFDF) (*p* < 0.05) with respect to the time in the central zone; however, with respect to rearing, no significant differences were detected between MEDF (300 mg/kg), AFDF (30 mg/kg) and DZP (2 mg/kg) (Table 4). In relation to grooming, MEDF (100 and 300 mg/kg) and AFDF (30 mg/kg) caused decreases of 58%, 77% and 70% (*p* < 0.001), respectively, compared with the vehicle (Table 4), and no statistically significant differences were detected among the treatments (MEDF, AFDF and DZP) (*p* < 0.05) (Table 4).

**Figure 4:**
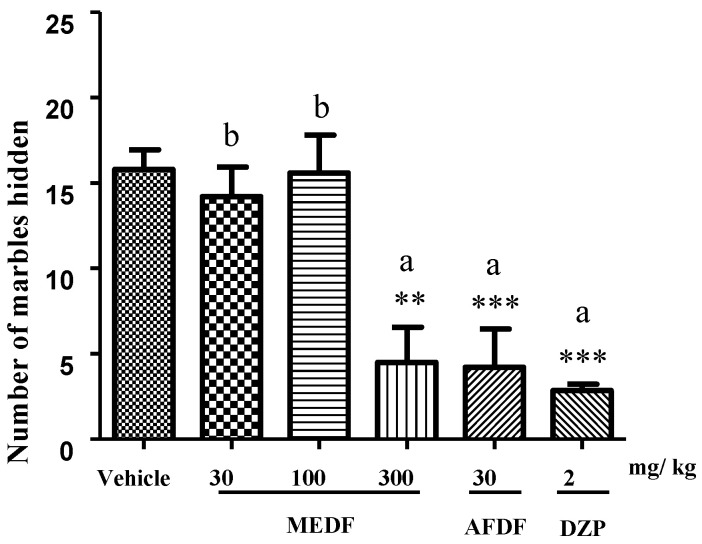
Marble burying test. Marbles were buried by mice treated with EMDF (30, 100, 300 mg/kg) and AFDF 30 mg/kg for 15 min on the tenth day of treatment. Values are presented as mean ± SEM. The symbol (*) denotes statistical difference from the vehicle, with *p* < 0.01 (**) and *p* < 0.001 (***) and (a, b) indicates the significant differences between treated groups (*p* < 0.05). The number of animals used in the experiments was n = 8 per group. Significance values were determined using ANOVA followed by Tukey multiple comparison test for the analysis.

Compared with the vehicle, the oral administration of MEDF (300 mg/kg) and AFDF (30 mg/kg) decreased the number of marbles hidden by 72% (*p* < 0.01) and 74% (*p* < 0.001), respectively (Figure 4). A comparison of the treatments revealed that DZP (2 mg/kg) (*p* < 0.05) did not significantly differ from MEDF (300 mg/kg) or AFDF (30 mg/kg) (Figure 4).

### 3.5. Memory and Learning—Anti-Amnesic Activity

**Figure 5:**
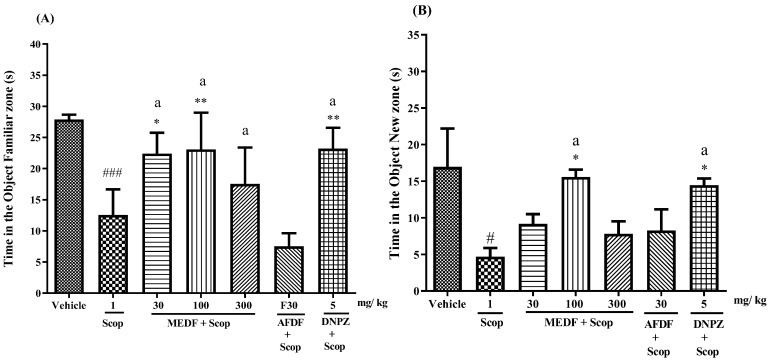
Object recognition test. (**A**) Time spent by the animal in the familiar object zone. (**B**) Time spent by the animal in the new object zone. Values are expressed as mean ± standard error of the mean (SEM). (^#^ *p* < 0.05 and ^###^ *p* < 0.001) statistically different from the vehicle compared with Scop group, and (*) statistically different from the group treated compared with Scop (* *p* < 0.05 and ** *p* < 0.01). (a) indicates no significant differences between treated groups (*p* < 0.05). Significance values were obtained through analysis of variance (ANOVA) and Tukey’s Multiple Comparison Test post-test.

The MEDF treatment (100 mg/kg) resulted in a preference for the novel object over the familiar object (Figure 5B). The preference of the novel object was 69% (*p* < 0.05) greater than that of Scop (1 mg/kg); similarly, the preference of DNPZ (5 mg/kg) was approximately 68% greater than that of Scop (1 mg/kg). A comparison between the treatments revealed that MEDF (100 mg/kg) and DZP (2 mg/kg) did not significantly differ in terms of novel objects (*p* < 0.05) or familiar objects (*p* < 0.01) (Figure 5A,B).

**Figure 6:**
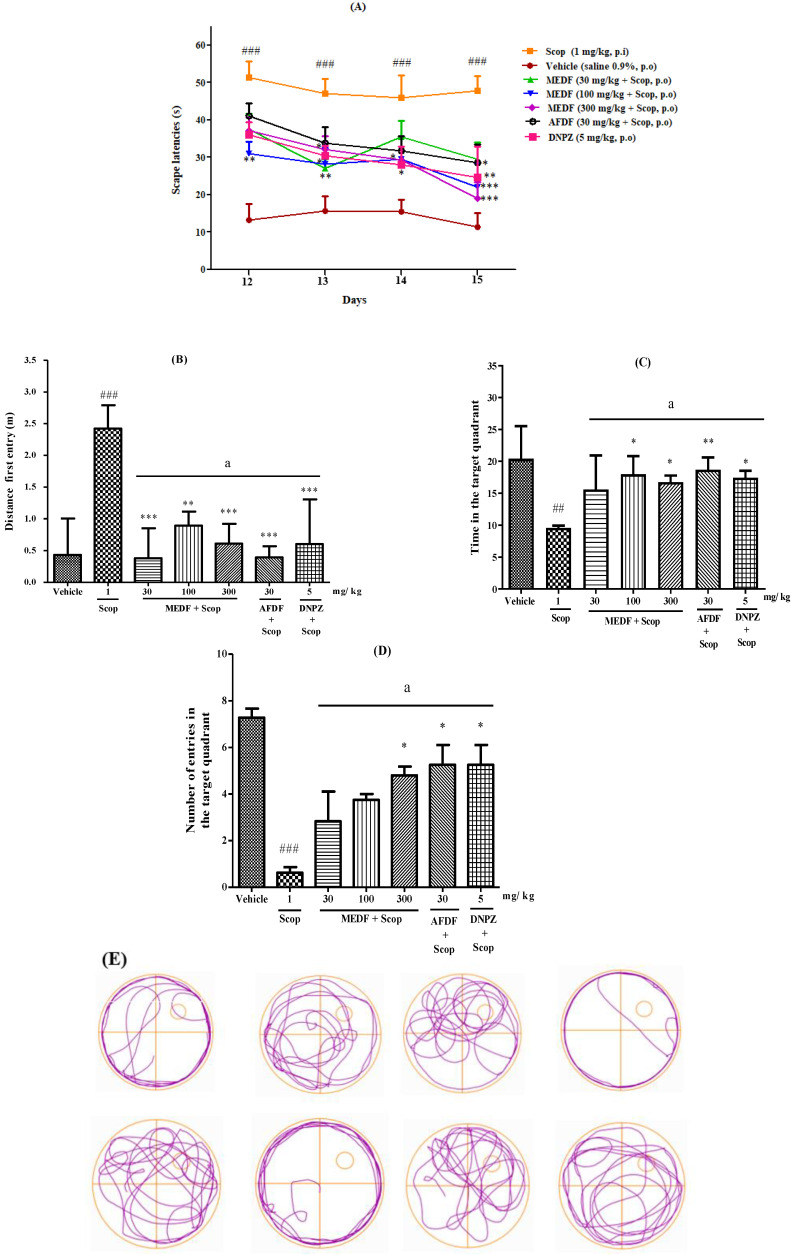
Effect of MEDF and AFDF on learning and memory in scopolamine-treated mice in the MWM test. (**A**) Escape latency of the acquisition trial over 4 days. (**B**) Distance covered since the first entry in the platform area. (**C**) The time the animal spent in the platform area. (**D**) The number of entries into the platform area. (**E**) Swimming trajectory performed by the group AFDF (30 mg/kg) during the test day (16th day, without the platform). Results were expressed as mean ± SEM (n = 8). (*) *p* < 0.05, (**) *p* < 0.01, (***) *p* < 0.001 versus the Scop-treated group; (##) *p* < 0.01, and (###) *p* < 0.001 versus the vehicle-Scop group. (a) indicates no significant differences between treated groups (*p* < 0.05). Significance values were obtained using analysis of variance (ANOVA) and Tukey’s multiple comparison test post-test.

Regarding long-term spatial learning capacity and memory (MWM), the results revealed that Scop (1 mg/kg) led to decreased memory performance (Figure 6A). The escape latency of the acquisition trials increased over the four days (12th to 15th day), indicating that the platform location ability of the Scop (47.80 s) was lower than that of the vehicle (11.39 s) (Figure 6A). Compared with Scop (47.80 s), MEDF (30, 100 and 300 mg/kg) and AFDF (30 mg/kg) on the 15th day of training (29.51 s, 21.93 s, 19.10 s and 28.53 s, respectively, effectively reduced the escape latency (Figure 6A).

The distance traveled from the first entry into the target quadrant on the test day (16th day) in the Scop (2.422 m) (*p* < 0.001) compared with the vehicle (0.434 m) group (Figure 6B) demonstrated a significant increase in distance traveled. In contrast, compared with Scop, MEDF (30 and 300 mg/kg) and AFDF (30 mg/kg) significantly decreased the distance traveled (0.381 and 0.610 m) (*p* < 0.001) and (0.393 m) (*p* < 0.001), respectively (Figure 6B). No statistically significant differences were detected among any of the treatments (MEDF, AFDF and DNPZ) (*p* < 0.05) (Figure 6B). Compared with the vehicle, Scop caused a significant decrease in time (9.6 s) (*p* < 0.01) in the target quadrant (20.43 s) (Figure 6C). Compared with Scop (9.6 s), AFDF (30 mg/kg) significantly increased the time spent in the target quadrant (19 s) (*p* < 0.01), whereas MEDF (100 and 300 mg/kg) increased the time spent in the target quadrant (18 s and 16 s) (*p* < 0.05) (Figure 6C), indicating an improvement in spatial memory formation in the treated animals. The number of entries into the target quadrant was lower in the Scop group (0.625) (*p* < 0.001) than in the vehicle group (7.0) (Figure 6D). Compared with Scop, MEDF (300 mg/kg) and AFDF (30 mg/kg) increased the number of entries into the target quadrant (4.8 and 5.25) (*p* < 0.05), respectively (Figure 6D). As shown, the latency to find the platform decreased in the AFDF (30 mg/kg) group during the acquisition phase (Figure 6E).

### 3.6. Biochemical Analysis—MDA and AChE

**Figure 7:**
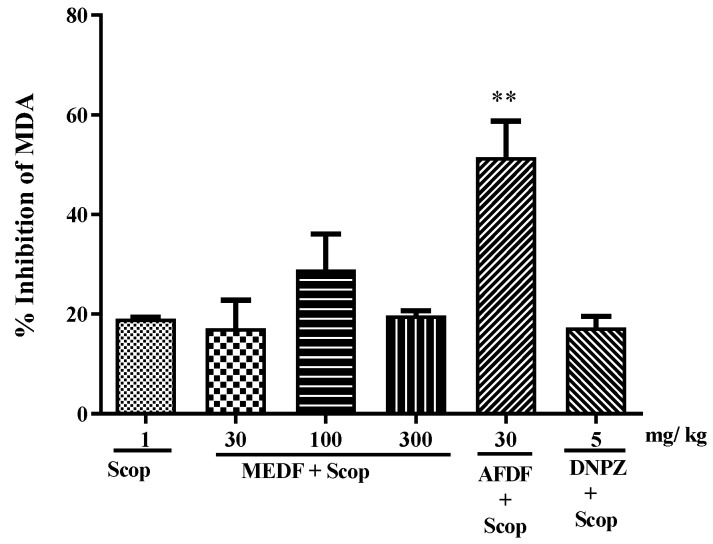
Effect of MEDF (30, 100 and 300 mg/kg) and AFDF (30 mg/kg) in MDA levels in brain mice after daily administration of Scop (11th to 16th days). (**) *p* < 0.01 versus Scop-treated group. Each bar represents mean ± SEM. Significance values were obtained using ANOVA and Tukey’s multiple comparison test post-test.

**Figure 8:**
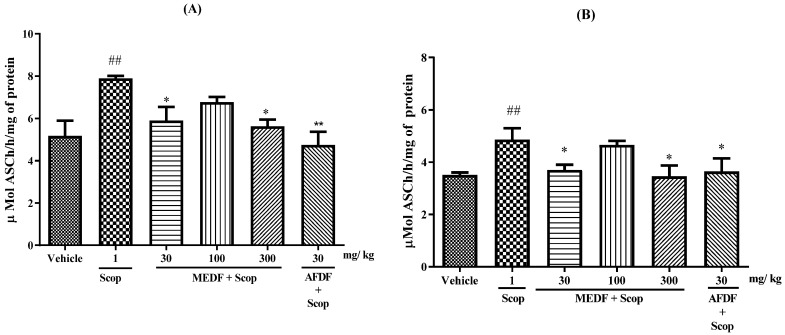
Effect of MEDF (30, 100 and 300 mg/kg) and AFDF (30 mg/kg) on the AChE activity in the cerebral cortex (**A**) and hippocampus (**B**). (*) *p* < 0.05, (**) *p* < 0.01 versus the Scop-treated group, (##) *p* < 0.01 versus the Scop-vehicle group. Each bar represents mean ± SEM. Significance values were obtained using ANOVA and Tukey’s multiple comparison test post-test.

The oral administration of AFDF (30 mg/kg) inhibited MDA levels by 51.51% (*p* < 0.01) (Figure 7). AChE activity was altered in both brain structures (Figure 8). After treatment with MEDF (30 and 300 mg/kg) or AFDF (30 mg/kg), AChE activity in the cortex was significantly lower (25 and 28% (*p* < 0.05), and 40% (*p* < 0.01), respectively) after 16 days of oral treatment than in the Scop group (Figure 8A). Compared with those in the Scop group, 24%, 29% and 25% (*p* < 0.05) of the hippocampus was affected by the oral administration of MEDF (30 and 300 mg/kg) or AFDF (30 mg/kg), respectively (Figure 8B).

### 3.7. Molecular Docking

**Figure 9:**
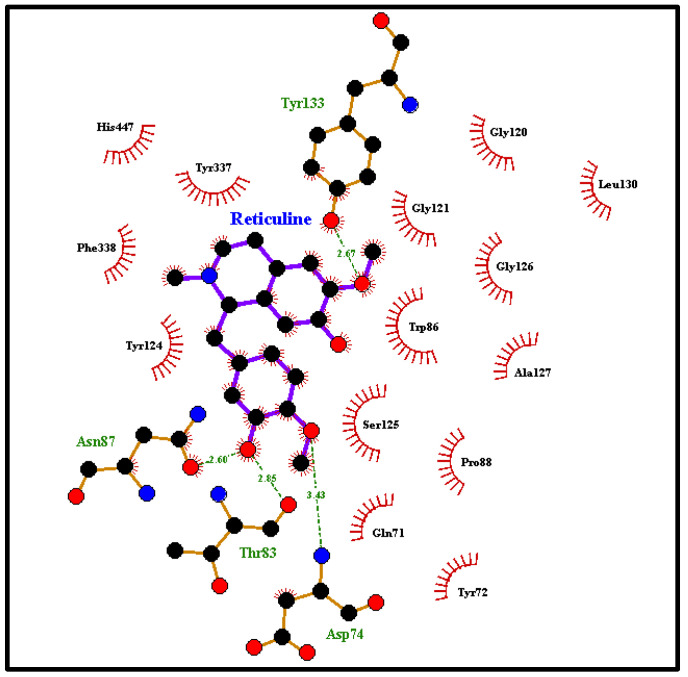
Docking interactions between the active residues site of the protein with the reticuline ligand.

**Figure 10:**
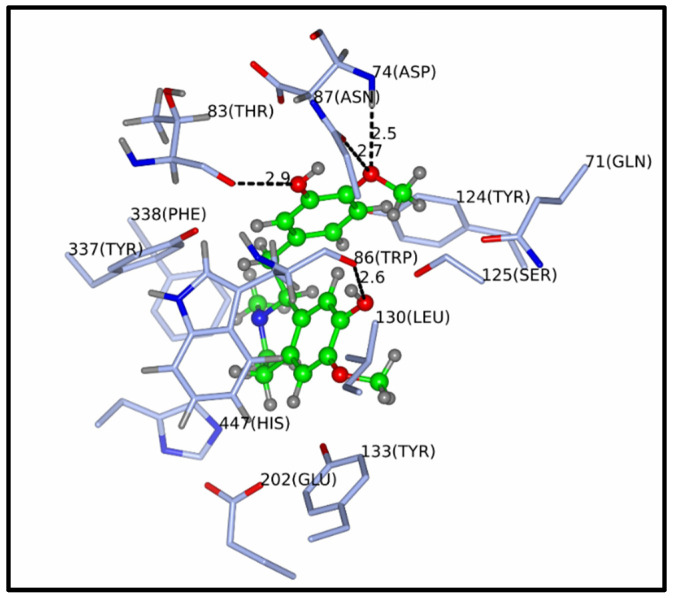
Overall view of the AChE subunit complexed with reticuline.

Figure 9 shows the reticuline link-type interactions and hydrogen bonds with Trp86, Asn87, Asp74 and Thr83 distances of 2.6, 2.5, 2.7 and 2.9 Å, respectively. In addition, there is a π-*stacking* interaction between the aromatic ring and the residue of Trp86 with the aromatic ring of the binder. In addition, van der Waals interactions can be observed between the cyclic portion of methylpiperidine and the aromatic ring of the Tyr337 residue (Figure 10).

## 4. Discussion

The present study provides the first evidence of the neurobehavioral effects of *D. furfuracea*, specifically its anxiety-like and anti-amnesic properties. These effects are attributed to the species’ rich phytochemical composition, particularly its alkaloid content, as well as the presence of reticulin. To the best of our knowledge, this is the first report evaluating the impact of *D. furfuracea* on neurobehavioral parameters, and these findings contribute to the understanding of the biological activities within the *Annonaceae* family.

First, we evaluated the contents of phenolic and polyphenolic compounds. MEDF presented high levels of condensed tannins and phenolic compounds, which prompted us to investigate their antioxidant activity via three different assays (free radical scavenging—DPPH and ABTS; lipoperoxidation—β-carotene). The DPPH assay is more applicable to hydrophobic systems, in which radicals dissolve in organic solvents while the ABTS assay is more applicable to hydrophilic and hydrophobic (lipophilic) systems. The β-carotene bleaching method evaluates the ability of a substance to prevent the oxidation of β-carotene, protecting it from the free radicals generated during the peroxidation of linoleic acid.

MEDF was the most active in the free radical scavenging (DPPH and ABTS) tests, which may be partly correlated with the high content of these constituents. The use of the solvent methanol for the preparation of MEDF is due to the efficient extraction of active compounds such as phenolics, polyphenolics, alkaloids and others, resulting in an extract with a higher concentration of compounds than other solvents, in addition to ensuring standardization and reproducibility, which corroborates those reported in our work [9,10]. Additionally, we suggest that this antioxidant action may also be related to the presence of alkaloids, which have been highlighted in studies of plants of this genus [18].

The DPPH reagent is a radical of organic nitrogen that is stable and violet in color and measures the ability of a substance to scavenge the DPPH radical, reducing it to hydrazine. When a substance that acts as a donor of hydrogen atoms is added to a solution of DPPH, hydrazine is obtained, with a change in color from violet (radical organic nitrogen, stable), which has absorption in the range of 515–520 nm, to pale yellow (DPPH radical reduction is monitored by the decrease in absorbance during the reaction). ABTS+ is a decolorization technique in which the radical is generated directly in a stable form prior to reacting with putative antioxidants, which involves the production of the blue/green ABTS+ chromophore through the reaction of ABTS with potassium persulfate, with absorption readings at 645, 734 and 815 nm. The evaluation of both methods is in accordance with the effects observed in the MEDF due to the presence of phenolics, polyphenolics and alkaloids. Oxidative stress is involved in several diseases, especially in the nervous system, such as AD.

Before conducting the behavioral tests, we performed a dereplication study via UHPLC–MS/MS and posterior docking simulations to support the biological tests. Reticuline was identified in AFDF by LC–MS/MS (Figure 2). The presence of the fragment at *m/z* = 192.102 [M^+^ − 137] is compatible with the literature data [36,37]. This fragment is formed by the induced connection break of the tyramine amino acid portion of the molecule and the loss of [C_8_H_9_O_2_]^+^, a characteristic of benzylisoquinoline. We can also suggest fragmentation at m/z = 177 [192-CH_3_]^+^ by the loss of a methyl group (Figure 2).

Reticuline is an alkaloid benzylisoquinoline precursor of the principal opium alkaloids thebaine, morphine and papaverine [38]. Neuropharmacological studies have been carried out with reticuline, which has been suggested to have potent effects on the central nervous system [39,40,41,42]. Reticuline has been shown to be related to the enzyme AChE, which is responsible for degrading the neurotransmitter acetylcholine; in vitro and in vivo studies have revealed that reticuline may modulate the activity of this enzyme, suggesting a potential therapeutic role in neurodegenerative conditions associated with cholinergic dysfunction [43,44].

During in vivo experimentation, the effects of chronically administered MEDF and AFDF were measured via different behavioral tests of neurocognitive disorders involving anxiety (open field test and marble burying) and learning and memory (object recognition test and MWM). Biochemical parameters (MDA and AChE) were also evaluated. After 10 days of treatment with MEDF or AFDF, fewer buried marbles were observed (Figure 4), indicating that the mice were not in a stressful situation, in which they were very agitated, or in a situation in which they felt threatened. Among the parameters evaluated in the open field test, MEDF (300 mg/kg) and AFDF (30 mg/kg) had anxiolytic effects (Table 4) because spontaneous movements between the center and periphery reduce rearing and grooming (exposure and exploratory activities).

On Day 11, the object recognition test (short-term memory) was carried out 1 h after Scop induction on the basis of exploratory activity (indicating the preservation of cognitive ability) when the mice were exposed to new objects. Only MEDF (100 mg/kg) significantly affected the time spent with the novel object (Figure 5B).

The MWM test was performed to assess behavioral patterns, such as deficits in long-term spatial learning capacity and memory (Figure 6). During the acquisition phase (12–15 days), all groups treated with MEDF (30, 100 or 300 mg/kg) and AFDF (30 mg/kg) presented reduced latency to find the platform, suggesting that the extract and alkaloidal fraction could minimize the damage of Scop to the cholinergic system, similar to DNPZ, a well-known anticholinesterase compound capable of increasing the levels of acetylcholine in the synaptic cleft and reversing failure of the cholinergic system (Figure 6A). On Day 16, MEDF (30 or 300 mg/kg) and AFDF (30 mg/kg) resulted in a shorter distance covered, and signaling increased memory formation (Figure 6B). MEDF (100 and 300 mg/kg) and AFDF (30 mg/kg) decreased the number of entries and time spent in the target quadrant, reinforcing memory formation (Figure 6C,D). These findings suggested that MEDF and AFDF could minimize the damage caused by scopolamine to the cholinergic system, identifying a candidate drug for the treatment of dementia.

A variety of compounds have been shown to be effective in protecting against amnesia induced by scopolamine, indicating that other mechanisms, in addition to the cholinergic system, are also involved in this model, including inhibitors of cholinesterase and antioxidant compounds. Oxidative stress has also been linked to the cognitive dysfunction associated with brain damage learning and memory, and it has been proposed that memory deficits are accompanied by changes in the indices/activities of oxidative stress markers [45,46,47]. Scop induces oxidative stress in nervous tissue, leading to an increase in MDA levels [48,49]. Our results showed that AFDF (30 mg/kg) was able to reduce the MDA level (Figure 6), indicating a restoration in the oxidative stress indices in the rat brain. The AFDF (30 mg/kg) reduces AChE activity in the frontal cortex and MEDF (30 or 300 mg/kg) and AFDF (30 mg/kg) in the hippocampus (Figure 8A,B). These regions are structures in the brain that are involved in learning, formation and memory storage (hippocampus) and in the encoding, storage and retrieval of memories in the cortex. In vitro studies using the methanolic extracts of leaves and seeds of *D. furfuracea* have demonstrated inhibitory effects on AChE activity [9].

In this study, we observed several limitations, such as significant improvements in behavioral outcomes across all the tested extract concentrations. However, the results did not follow a clear dose-dependent pattern.

To our knowledge, this is the first study that reveals the neuroprotective effect on chemically induced amnesia and anxiolytic effects in mice. However, the effect of this action on *D. furfuracea* is still unknown. Previous studies have shown that analogous results, such as the ethanolic extract of *Xylopia aethiopica* fruit, improved exploratory learning and recognition memory, as well as spatial working, recognition and reference memories, in behavioral tests in Annonaceae species [50]. Extracts from *X. parviflora* fruits increased memory; improved locomotion, cholinesterase activities and ion homeostasis and stabilized brain oxidative stress levels [51].

In the present study, our hypothesis is that this action may be associated with the presence of alkaloids, such as reticuline. Alkaloids reversibly inhibit AChE and improve cholinergic neurotransmission, similar to most Annonaceae alkaloids, which act on cognitive disorders [50,51,52,53]. The alkaloids present in *D. furfuracea*, such as anonaine and asimilobine, have antidepressant effects; xilopine and duguetine have sedative effects and reticuline has a CNS effect [20,54,55,56].

To explore the mode of interaction of reticuline, a compound identified in the extract of *D. furfuracea*, a molecular docking assay was performed, preferably for mAChE (since the inhibitory activity data used in the present study were related to this enzyme) to assess the enzyme–inhibitor interactions and propose a bonding model based on the experimental results. Reticuline was anchored to the AChE binding site via link-type interactions, hydrogen bonds and van der Waals interactions (Figure 9 and Figure 10). The hydrolysis mechanism of acetylcholine involves mainly the catalytic triad residues (Ser200, His440 and Glu327). Upon analysis of the enzyme-substrate complex, one can observe that the reticuline is distant from the catalytic triad residues, decreasing the possibility of the enzyme inhibition, corroborating with the experimental data, which showed that AFDF was involved in AChE activity (40%) in the cortex. However, even when this reticuline effect is observed, this task is further complicated by the fact that the overall activity of extracts of medicinal plants is possibly a result of the combined action of multiple compounds with synergistic, additive or antagonistic activity.

Studies on *D. furfuracea* extracts reveal a complex biological profile, showing both cytotoxic effects in certain contexts and indications of safety or protective properties in others. One set of findings indicates that while *D. furfuracea* leaf extract exhibits cytotoxicity, it does not display genotoxic effects [11]. In studies with Drosophila melanogaster, a seven-day exposure to a hydroalcoholic leaf extract of *D. furfuracea* resulted in toxicity associated with oxidative stress, highlighting its potential to disrupt cellular processes with prolonged exposure [14]. Conversely, the research on lyophilized *D. furfuracea* leaf extract demonstrated antigenotoxic and anticytotoxic effects, suggesting a possible protective role under specific conditions [13]. Further studies found that an aqueous extract of *D. furfuracea* exhibited toxic effects in female rats during pregnancy [12]. In contrast, in the present study, MEDF did not induce detectable toxicity in terms of clinical signs, blood profiles, organ morphology or cellular integrity within the tested dosage range. It is important to note that this study differs from those cited above, as it involved in vivo assays with distinct doses and treatment durations.

## 5. Conclusions

In conclusion, MEDF and AFDF of *D. furfuracea* leaves demonstrated a memory-enhancing effect in mice. This was accomplished by suppressing the action of AChE and mitigating oxidative stress. The presence of the alkaloid reticuline could be responsible, at least in part, for the observed effects, in addition to the synergistic effect of the high content of polyphenolic compounds observed. Furthermore, this study demonstrated the absence of toxicity of MEGV in vivo, which can contribute to the safe use of this species. Further studies are needed to optimize dosing to establish a more consistent and reliable dose–response relationship and define the mechanism of action.

## Figures and Tables

**Table 1:** Antioxidant activity of methanolic extract (MEDF) and alkaloid-fractions (AFDF) of *D. furfuracea* leaves.

Samples	DPPH	ABTS	β-Carotene/Linoleic Acid
	IC_50_ (µg/mL)		
MEDF	18.10 ± 1.70	10.41 ± 1.69	77.47 ± 9.92
AFDF	24.22 ± 0.80	32.38 ± 2.12	108.44 ± 3.56
BHT	9.89 ± 2.90	5.24 ± 2.67	13.03 ± 2.67

The values represent the means of three measurements ± standard deviation.

**Table 2:** Body weight gain, food and water consumption of female Swiss mice treated with MEDF.

	1st Day	7th Day	14th Day	21th Day	28th Day
	Body Weight (g)				
Vehicle	26.33 ± 0.95	25.50 ± 0.56	28.00 ± 1.26	28.16 ± 0.94	27.83 ± 0.70
MEDF (30 mg/kg)	25.66 ± 0.55	25.16 ± 0.70	26.50 ± 0.99	27.20 ± 0.48	28.40 ± 0.51
MEDF (100 mg/kg)	25.83 ± 0.91	26.00 ± 0.70	27.50 ± 0.56	27.66 ± 0.71	27.16 ± 0.70
MEDF (300 mg/kg)	26.66 ± 0.76	26.16 ± 0.79	26.33 ± 0.76	26.83 ± 0.70	26.66 ± 0.71
	Food intake (g/Day)				
Vehicle	-	15.50 ± 0.33	26.33 ± 1.22	28.50 ± 0.56	33.17 ± 0.86
MEDF (30 mg/kg)	-	13.67 ± 0.68	23.00 ± 0.46	27.60 ± 0.33	34.80 ± 0.73
MEDF (100 mg/kg)	-	15.33 ± 0.63	24.33 ± 0.88	25.83 ± 0.35	31.33 ± 0.88
MEDF (300 mg/kg)	-	14.33 ± 0.51	20.83 ± 1.45	24.00 ± 0.79	30.67 ± 0.64
	Water intake (mL/Day)				
Vehicle	-	31.67 ± 0.30	70.00 ± 1.43	65.00 ± 1.38	81.67 ± 5.22
MEDF (30 mg/kg)	-	31.67 ± 0.28	76.67 ± 2.43	52.00 ± 2.44	60.00 ± 3.34
MEDF (100 mg/kg)	-	31.67 ± 0.44	60.00 ± 4.35	64.17 ± 3.33	53.33 ± 3.43
MEDF (300 mg/kg)	-	36.67 ± 2.24	46.67 ± 4.32	48.33 ± 4.67	48.33 ± 6.43

Values correspond to mean ± SEM (n = 6/group). No significant difference (*p* < 0.05) was found in relation to vehicle.

**Table 3:** Relative organ weights, hematological and biochemical values of female Swiss mice treated with an MEDF.

			MEDF		
Parameters	Control	Vehicle Tween 5%	30 mg/kg	100 mg/kg	300 mg/kg
Organs (g)					
Brain	0.44 ± 0.01	0.47 ± 0.01	0.47 ± 0.01	0.46 ± 0.00	0.46 ± 0.00
Heart	0.14 ± 0.00	0.14 ± 0.00	0.14 ± 0.00	0.13 ± 0.00	0.13 ± 0.00
Lung	0.21 ± 0.00	0.24 ± 0.01	0.25 ± 0.01	0.23 ± 0.01	0.22 ± 0.00
Liver	1.18 ± 0.03	1.15 ± 0.03	1.18 ± 0.03	1.13 ± 0.06	1.05 ± 0.03
Kidney	0.38 ± 0.01	0.38 ± 0.01	0.39 ± 0.01	0.37 ± 0.00	0.33 ± 0.00
Spleen	0.15 ± 0.01	0.13 ± 0.00	0.14 ± 0.01	0.14 ± 0.01	0.12 ± 0.00
Biochemical					
CBG (mg/dL)	104.25 ± 2.46	98.33 ± 4.05	104.33 ± 5.36	95.00 ± 7.23	129.16 ± 4.46
CHL (mg/dL)	56.07 ± 3.01	47.00 ± 4.44	69.80 ± 5.10	79.86 ± 10.06	67.60 ± 8.10
ALB (mg/dL)	25.09 ± 1.67	25.90 ± 2.57	30.32 ± 2.51	26.86 ± 3.37	33.24 ± 1.07
UR (mg/dL)	41.45 ± 1.31	40.83 ± 2.92	37.26 ± 1.94	29.70 ± 1.80	39.60 ± 7.60
ALT (U/L)	38.45 ± 2.54	40.22 ± 6.15	32.50 ± 5.28	31.46 ± 6.73	55.45 ± 27.45
AST (U/L)	165.05 ± 2.55	124.50 ± 2.51	150.60 ± 31.05	258.23 ± 52.77	157.10 ± 0.00
Erythogram					
RBC (×10^6^/µL)	8.53 ± 0.25	9.24 ± 0.24	6.80 ± 0.05	9.69 ± 0.21	9.70 ± 0.27
HCT (%)	41.27 ± 1.03	48.12 ± 1.61	33.50 ± 0.20	50.28 ± 1.44	51.06 ± 0.81
HGB (g/dL)	12.90 ± 0.34	14.42 ± 0.44	10.30 ± 0.10	14.90 ± 0.39	15.48 ± 0.23
MCV (fL)	48.37 ± 0.30	51.57 ± 0.33	49.25 ± 0.65	51.80 ± 0.46	52.68 ± 0.91
MCH (pg)	15.12 ± 0.04	15.45 ± 0.06	15.15 ± 0.25	15.36 ± 0.17	15.98 ± 0.28
MCHC (g/dL)	31.25 ± 0.08	29.80 ± 0.22	30.75 ± 0.15	29.66 ± 0.17	30.30 ± 0.22
RDW-SD (fL)	28.07 ± 0.36	27.45 ± 0.12	27.70 ± 0.30	28.64 ± 0.48	28.18 ± 0.33
RDW-CV (%)	14.65 ± 0.40	13.15 ± 0.29	12.65 ± 0.25	15.16 ± 0.74	14.10 ± 0.44
Plaquetogram					
PLT (×10^3^/µL)	928.33 ± 33.31	982.50 ± 39.73	656.00 ± 82.00	977.20 ± 54.94	1034.00 ±84.79
MPV (fL)	6.60 ± 0.14	6.62 ± 0.12	6.50 ± 0.10	6.56 ± 0.10	6.50 ± 0.07
Leukogram					
WBC (×10^3^/µL)	3.45 ± 0.39	3.70 ± 0.04	2.65 ± 0.25	4.30 ± 0.49	4.32 ± 0.34
LYM (%)	38.75 ± 1.10	34.50 ± 4.40	30.00 ± 0.00	17.00 ± 5.26	19.60 ± 5.39
EOS (%)	1.00 ± 0.00	1.00 ± 0.00	1.00 ± 0.00	1.20 ± 0.20	1.00 ± 0.00
MON (%)	2.75 ± 0.47	3.00 ± 0.91	2.50 ± 1.50	2.20 ± 0.49	1.60 ± 0.60
NEUT (%)	56.00 ± 1.22	60.50 ± 5.80	66.50 ± 1.50	79.60 ± 4.95	77.80 ± 5.72

The creatinine (CR) showed values below the detection limits (<0.2 mg/dL). Capillary blood glucose (CBG); cholesterol (CHL); albumin (ALB); urea (UR); alanine transaminase (ALT); aspartate transaminase (AST); red blood cell (RBC); hematocrit (HCT); mean corpuscular volume (MCV); mean corpuscular hemoglobin (MCH); mean corpuscular hemoglobin concentration (MCHC); red blood dimension width–standard deviation (RDW-SD); red cell distribution width—coefficient variation (RDW-CV); number of platelets (PLT); mean platelet volume (MPV); number of white blood cells (WBC); lymphocytes (LYM); eosinophils (EOS); monocyte (MON) and neutrophils (NEUT). Values correspond to mean ± SEM (n = 6/group). No significant difference (*p* > 0.05) was found in relation to control.

**Table 4:** MEDF (30, 100 or 300 mg/kg) and AFDF (30 mg/kg) on the anxiolytic effect, assessed via the open field test.

Treatments	Time in the Zone Central (s)	Number of Rearing	Number of Grooming
Vehicle	8.88 ± 1.19	13.00 ± 1.78	7.50 ± 1.04
MEDF (30 mg/kg)	24.84 ± 2.63 ^b^	10.57 ± 1.52 ^b^	3.63 ± 0.53 **^a^
MEDF (100 mg/kg)	31.4 ± 4.82 ^b^	12.14 ± 2.32 ^b^	3.13 ± 0.39 ***^a^
MEDF (300 mg/kg)	38.57 ± 5.44 *^b^	4.28 ± 0.71 **^a^	1.75 ± 0.36 ***^a^
AFDF (30 mg/kg)	45.45 ± 10.32 **^b^	4.14 ± 0.59 **^a^	2.28 ± 0.74 ***^a^
DZP (2 mg/kg)	61.74 ± 1.74 ***^a^	4.0 ± 1.06 **^a^	2.67 ± 0.76 ***^a^

Values are reported as mean ± standard error of the mean (SEM) (n = 8), with significant differences indicated as follows: *p* < 0.05 (*), *p* < 0.01 (**) and *p* < 0.001 (***) statistically different from the vehicle-treated groups (in the column) and (a, b) indicates the significant differences between treated groups (*p* < 0.05). Significance values were determined using ANOVA followed by Tukey multiple comparison test for the analysis.

## Data Availability

All data are available upon request from the corresponding author.

## Supplementary Materials

The following supporting information can be downloaded at: https://www.mdpi.com/article/10.3390/biology13120981/s1, Figure S1: TLC (CHCl_3_: MeOH at 70%) by Dragendorff’s reagent to confirm that AFDF was positive for alkaloids.
