# Supplementary material for: Duguetia furfuracea (A.ST. Hil.) Saff.: Neuroprotective Effect on Chemically Induced Amnesia, Anxiolytic Effects and Preclinical Safety Evaluation in Mice
Source: Biology (Basel). 2024 Nov 27;13(12):981. doi: 10.3390/biology13120981 (PMC11726886; doi:10.3390/biology13120981)
Supplement: Supplementary file 1 [file biology-13-00981-s001.zip › biology-3269134-supplementary.pdf]

Supplementary material

Duguetia furfuracea **(A.ST. Hil.) Saff.: Neuroprotective effect on chemically induced amnesia, anxiolytic effects and preclinical safety evaluation in mice**

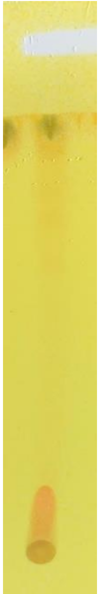

Figure S1. TLC ($CHCl_3$: MeOH at 70%) by Dragendorff's reagent to confirm that AFDF was positive for alkaloids.
